# Crystal structure of 2-{[2-meth­oxy-5-(tri­fluoro­meth­yl)phen­yl]iminomethyl}-4-nitro­phenol

**DOI:** 10.1107/S2056989015010129

**Published:** 2015-06-13

**Authors:** Nevzat Karadayı, Songül Şahin, Yavuz Köysal, Emine Coşkun, Orhan Büyükgüngör

**Affiliations:** a Yeşilyurt Demir Celik Higher Vocational School, Ondokuz Mayıs University, TR-55330 Tekkeköy-Samsun, Turkey; b Department of Chemistry, Faculty of Arts and Sciences, Ondokuz Mayıs University, TR-55139 Samsun, Turkey; c Department of Physics, Faculty of Arts and Sciences, Ondokuz Mayıs University, TR-55139 Samsun, Turkey

**Keywords:** crystal structure, Schiff base, hydrogen bonding

## Abstract

In the title compound, C_15_H_11_F_3_N_2_O_4_, the N=C bond of the central imine group adopts an *E* conformation. The dihedral angle between two benzene rings is 6.2 (2)°. There is an intra­molecular bifurcated O—H⋯(N,O) hydrogen bond with *S*(6) and *S*(9) ring motifs. In the crystal, mol­ecules are linked by C—H⋯O hydrogen bonds into a helical chain along the 3_1_ screw axis parallel to *c*. The –CF_3_ group shows rotational disorder over two sites, with occupancies of 0.39 (2) and 0.61 (2).

## Related literature   

For photochromic, thermochromic and biological applications of related Schiff base compounds, see: Hadjoudis *et al.* (1987 ▸); Santos *et al.* (2001 ▸); Tarafder *et al.* (2002 ▸). For related structures, see: Faridbod *et al.* (2008 ▸); Karadayı *et al.* (2003 ▸, 2006 ▸, 2013 ▸); Raja *et al.* (2008 ▸).
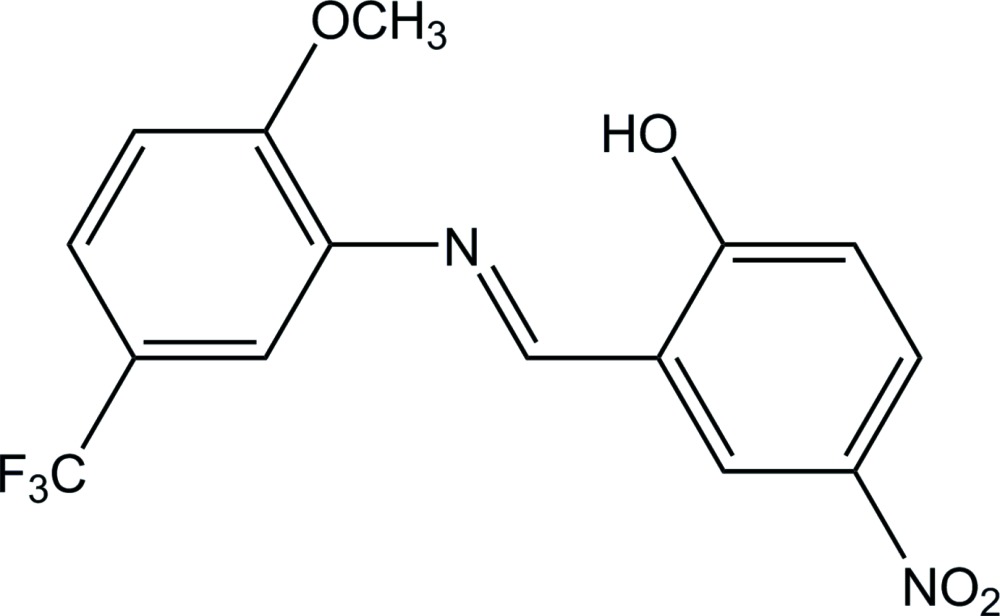


## Experimental   

### Crystal data  

C_15_H_11_F_3_N_2_O_4_
*M* *_r_* = 340.26
Trigonal, 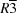

*a* = 33.0327 (16) Å
*c* = 7.1523 (3) Å
*V* = 6758.7 (5) Å^3^
*Z* = 18
Mo *K*α radiation
μ = 0.13 mm^−1^
*T* = 296 K
0.67 × 0.25 × 0.04 mm

### Data collection  

Stoe IPDS 2 diffractometer
Absorption correction: integration (*X-RED32*; Stoe & Cie, 2002 ▸) *T*_min_ = 0.951, *T*_max_ = 0.994
16356 measured reflections
2958 independent reflections
1380 reflections with *I* > 2σ(*I*)
*R*_int_ = 0.140

### Refinement  

*R*[*F* ^2^ > 2σ(*F* ^2^)] = 0.084
*wR*(*F* ^2^) = 0.126
*S* = 1.07
2958 reflections
246 parameters
H-atom parameters constrained
Δρ_max_ = 0.15 e Å^−3^
Δρ_min_ = −0.19 e Å^−3^

### 

Data collection: *X-AREA* (Stoe & Cie, 2002 ▸); cell refinement: *X-AREA*; data reduction: *X-RED32* (Stoe & Cie, 2002 ▸); program(s) used to solve structure: *SHELXS97* (Sheldrick, 2008 ▸); program(s) used to refine structure: *SHELXL97* (Sheldrick, 2008 ▸); molecular graphics: *ORTEP-3 for Windows* (Farrugia, 2012 ▸); software used to prepare material for publication: *WinGX* (Farrugia, 2012 ▸).

## Supplementary Material

Crystal structure: contains datablock(s) I. DOI: 10.1107/S2056989015010129/is5398sup1.cif

Structure factors: contains datablock(s) I. DOI: 10.1107/S2056989015010129/is5398Isup2.hkl

Supporting information file. DOI: 10.1107/S2056989015010129/is5398Isup3.cml

**Fig. 1.**
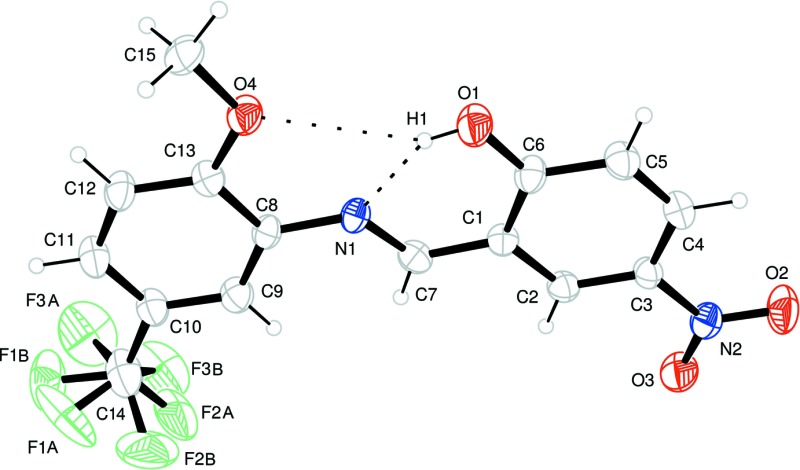
An ORTEP drawing of the title compound showing the atomic numbering scheme. Displacement ellipsoids of non-H atoms are shown at the 20% probability level. Hydrogen bonds are indicated by dashed lines.

ORTEP . DOI: 10.1107/S2056989015010129/is5398fig1.tif
An *ORTEP* drawing of the title compound showing the atomic numbering scheme. Displacement ellipsoids of non-H atoms are shown at the 20% probability level. Hydrogen bonds are indicated by dashed lines.

CCDC reference: 1402674

Additional supporting information:  crystallographic information; 3D view; checkCIF report

## Figures and Tables

**Table 1:** Hydrogen-bond geometry (, )

*D*H*A*	*D*H	H*A*	*D* *A*	*D*H*A*
O1H1N1	0.82	1.84	2.571(4)	148
O1H1O4	0.82	2.76	3.468(4)	146
C7H7O2^i^	0.93	2.55	3.476(7)	176
C9H9O2^i^	0.93	2.46	3.378(6)	169

Symmetry code: (i) 
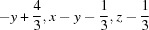
.

## supplementary crystallographic information

### S1. Comment

Schiff base compounds can be classified by their photochromic and thermochromic characteristics (Hadjoudis *et al.*, 1987). Schiff bases are potentially biologically active compounds and the antifungal, anticancer, anticonvulsant, diuretic and cytotoxic activities have been reported. For the development of bacteriostatic activity, it is believed that the presence of a nitro group in the *p*-position is an important condition (Tarafder *et al.*, 2002; Santos *et al.*, 2001). In this study we report the structure of the title compound (I).

The N1═C7 bond length is 1.295 (5) Å, approximately equal to previously reported C═N bond lengths (Karadayı *et al.*, 2003; Faridbod *et al.*, 2008; Karadayı *et al.*, 2013). The geometric parameters in (I) are comparable with the similar reported structures (Raja *et al.*, 2008; Karadayı *et al.*, 2006). The dihedral angle between the aromatic rings (C1–C6) and (C8–C13) is 6.2 (2)°. The CF_3_ group showed rotational disorder. The site occupancy factors are 0.39 (2) and 0.61 (2) for F1A–F3A and F1B–F3B, respectively. An intramolecular bifurcated O—H···(N, O) hydrogen bond is observed (Table 1 and Fig. 1).

### S2. Experimental

The title compound was prepared by refluxing a mixture of a solution containing 2-hydroxy-5-nitrobenzaldehyde (0.014 g, 0.082 mmol) and a solution containing 2-methoxy-5-(trifluoromethyl)aniline (0,016 g, 0.082 mmol) in 20 ml ethanol. The reaction mixture was stirred for 1 h under reflux. Single crystals suitable for X-ray analysis were obtained from an ethanol solution by slow evaporation (yield 54%, *m*.*p*. 475–477 K).

### S3. Refinement

All H atoms were placed in calculated positions and refined using a riding model, with C—H = 0.93 or 0.96 Å and O—H = 0.82 Å. The isotropic displacement parameters of the H atoms were fixed at 1.2*U*_eq_(C) or 1.5*U*_eq_(O, C_methyl_). The CF_3_ group showed rotational disorder. The site occupancy factors are 0.39 (2) and 0.61 (2) for F1A–F3A and F1B–F3B, respectively.

### Crystal data

**Table:**

C_15_H_11_F_3_N_2_O_4_	*D*_x_ = 1.505 Mg m^−^^3^
*M**_r_* = 340.26	Mo *K*α radiation, λ = 0.71073 Å
Trigonal, *R*3	Cell parameters from 10608 reflections
Hall symbol: -R 3	θ = 2.1–28.0°
*a* = 33.0327 (16) Å	µ = 0.13 mm^−^^1^
*c* = 7.1523 (3) Å	*T* = 296 K
*V* = 6758.7 (5) Å^3^	Needle, light brown
*Z* = 18	0.67 × 0.25 × 0.04 mm
*F*(000) = 3132	

### Data collection

**Table:**

Stoe IPDS 2 diffractometer	2958 independent reflections
Radiation source: fine-focus sealed tube	1380 reflections with *I* > 2σ(*I*)
Graphite monochromator	*R*_int_ = 0.140
ω scans	θ_max_ = 26.0°, θ_min_ = 2.1°
Absorption correction: integration (*X-RED32*; Stoe & Cie, 2002)	*h* = −40→40
*T*_min_ = 0.951, *T*_max_ = 0.994	*k* = −39→40
16356 measured reflections	*l* = −8→8

### Refinement

**Table:**

Refinement on *F*^2^	Primary atom site location: structure-invariant direct methods
Least-squares matrix: full	Secondary atom site location: difference Fourier map
*R*[*F*^2^ > 2σ(*F*^2^)] = 0.084	Hydrogen site location: inferred from neighbouring sites
*wR*(*F*^2^) = 0.126	H-atom parameters constrained
*S* = 1.07	*w* = 1/[σ^2^(*F*_o_^2^) + (0.0292*P*)^2^] where *P* = (*F*_o_^2^ + 2*F*_c_^2^)/3
2958 reflections	(Δ/σ)_max_ = 0.001
246 parameters	Δρ_max_ = 0.15 e Å^−^^3^
0 restraints	Δρ_min_ = −0.19 e Å^−^^3^

### Special details

**Table:**

Geometry. All e.s.d.'s (except the e.s.d. in the dihedral angle between two l.s. planes) are estimated using the full covariance matrix. The cell e.s.d.'s are taken into account individually in the estimation of e.s.d.'s in distances, angles and torsion angles; correlations between e.s.d.'s in cell parameters are only used when they are defined by crystal symmetry. An approximate (isotropic) treatment of cell e.s.d.'s is used for estimating e.s.d.'s involving l.s. planes.
Refinement. Refinement of *F*^2^ against ALL reflections. The weighted *R*-factor *wR* and goodness of fit *S* are based on *F*^2^, conventional *R*-factors *R* are based on *F*, with *F* set to zero for negative *F*^2^. The threshold expression of *F*^2^ > σ(*F*^2^) is used only for calculating *R*-factors(gt) *etc*. and is not relevant to the choice of reflections for refinement. *R*-factors based on *F*^2^ are statistically about twice as large as those based on *F*, and *R*- factors based on ALL data will be even larger.

### Fractional atomic coordinates and isotropic or equivalent isotropic displacement parameters (Å^2^)

**Table:**

	*x*	*y*	*z*	*U*_iso_*/*U*_eq_	Occ. (<1)
O4	0.76299 (10)	0.04667 (9)	0.2585 (4)	0.0569 (8)	
O1	0.76808 (9)	0.14868 (10)	0.4121 (5)	0.0647 (9)	
H1	0.7798	0.1330	0.3798	0.097*	
N1	0.83251 (11)	0.12822 (11)	0.3519 (4)	0.0443 (8)	
N2	0.90475 (14)	0.32508 (12)	0.6373 (5)	0.0538 (9)	
C7	0.86313 (14)	0.17045 (14)	0.4002 (5)	0.0473 (10)	
H7	0.8947	0.1797	0.3969	0.057*	
C1	0.84911 (13)	0.20297 (13)	0.4583 (5)	0.0417 (10)	
O3	0.94606 (11)	0.33571 (11)	0.6341 (5)	0.0724 (10)	
C6	0.80087 (14)	0.18987 (14)	0.4640 (6)	0.0488 (11)	
C4	0.82253 (15)	0.26645 (15)	0.5825 (6)	0.0529 (11)	
H4	0.8141	0.2879	0.6241	0.063*	
C2	0.88280 (14)	0.24797 (13)	0.5163 (6)	0.0452 (10)	
H2	0.9143	0.2568	0.5131	0.054*	
C3	0.86965 (13)	0.27894 (13)	0.5775 (6)	0.0429 (10)	
C13	0.80545 (15)	0.04950 (14)	0.2514 (6)	0.0463 (10)	
C10	0.89519 (15)	0.06269 (15)	0.2580 (6)	0.0549 (12)	
O2	0.89212 (12)	0.35192 (11)	0.6969 (5)	0.0788 (10)	
C11	0.85770 (17)	0.01995 (16)	0.2083 (6)	0.0614 (13)	
H11	0.8627	−0.0045	0.1772	0.074*	
C5	0.78900 (15)	0.22311 (14)	0.5269 (6)	0.0544 (11)	
H5	0.7578	0.2152	0.5303	0.065*	
C9	0.88791 (15)	0.09902 (15)	0.3038 (6)	0.0532 (11)	
H9	0.9132	0.1278	0.3373	0.064*	
C8	0.84359 (13)	0.09318 (13)	0.3005 (5)	0.0424 (10)	
C12	0.81262 (16)	0.01297 (14)	0.2039 (6)	0.0568 (12)	
H12	0.7876	−0.0159	0.1695	0.068*	
C15	0.72262 (15)	0.00229 (14)	0.2147 (6)	0.0630 (13)	
H15A	0.6950	0.0048	0.2240	0.094*	
H15B	0.7205	−0.0209	0.3011	0.094*	
H15C	0.7254	−0.0066	0.0897	0.094*	
C14	0.9427 (2)	0.0689 (2)	0.2677 (12)	0.0853 (18)	
F1A	0.9456 (8)	0.0369 (9)	0.340 (5)	0.142 (14)	0.39 (2)
F2A	0.9748 (7)	0.1072 (7)	0.317 (7)	0.158 (16)	0.39 (2)
F3A	0.9570 (6)	0.0665 (12)	0.074 (2)	0.152 (9)	0.39 (2)
F1B	0.9474 (5)	0.0345 (4)	0.221 (3)	0.136 (9)	0.61 (2)
F2B	0.9590 (5)	0.0783 (9)	0.4489 (18)	0.156 (7)	0.61 (2)
F3B	0.9727 (4)	0.1064 (6)	0.181 (2)	0.128 (7)	0.61 (2)

### Atomic displacement parameters (Å^2^)

**Table:**

	*U*^11^	*U*^22^	*U*^33^	*U*^12^	*U*^13^	*U*^23^
O4	0.0435 (17)	0.0424 (17)	0.077 (2)	0.0155 (15)	0.0007 (15)	−0.0058 (15)
O1	0.0437 (17)	0.048 (2)	0.097 (3)	0.0190 (16)	0.0001 (17)	−0.0140 (17)
N1	0.045 (2)	0.0307 (18)	0.050 (2)	0.0140 (17)	−0.0002 (17)	−0.0023 (15)
N2	0.057 (3)	0.040 (2)	0.059 (2)	0.021 (2)	−0.0016 (19)	−0.0045 (18)
C7	0.040 (2)	0.051 (3)	0.051 (3)	0.024 (2)	0.005 (2)	0.006 (2)
C1	0.036 (2)	0.040 (2)	0.046 (2)	0.017 (2)	0.0036 (18)	0.0010 (19)
O3	0.047 (2)	0.058 (2)	0.100 (3)	0.0173 (17)	−0.0097 (18)	−0.0190 (18)
C6	0.046 (3)	0.035 (2)	0.058 (3)	0.014 (2)	0.005 (2)	−0.002 (2)
C4	0.058 (3)	0.049 (3)	0.061 (3)	0.034 (2)	0.004 (2)	0.001 (2)
C2	0.039 (2)	0.044 (3)	0.051 (3)	0.019 (2)	−0.0017 (19)	−0.0007 (19)
C3	0.043 (3)	0.035 (2)	0.051 (3)	0.020 (2)	−0.0026 (19)	0.0000 (19)
C13	0.047 (3)	0.043 (3)	0.048 (3)	0.021 (2)	0.008 (2)	0.008 (2)
C10	0.051 (3)	0.048 (3)	0.069 (3)	0.027 (2)	0.006 (2)	0.001 (2)
O2	0.081 (2)	0.0451 (19)	0.111 (3)	0.032 (2)	0.003 (2)	−0.0172 (19)
C11	0.065 (3)	0.047 (3)	0.079 (4)	0.033 (3)	0.006 (3)	0.000 (2)
C5	0.041 (3)	0.051 (3)	0.070 (3)	0.022 (2)	0.005 (2)	0.000 (2)
C9	0.049 (3)	0.044 (3)	0.062 (3)	0.020 (2)	0.002 (2)	0.002 (2)
C8	0.044 (3)	0.031 (2)	0.046 (2)	0.014 (2)	0.0038 (19)	0.0014 (18)
C12	0.058 (3)	0.035 (2)	0.071 (3)	0.019 (2)	0.001 (2)	−0.003 (2)
C15	0.046 (3)	0.045 (3)	0.081 (3)	0.010 (2)	−0.008 (2)	−0.009 (2)
C14	0.060 (4)	0.059 (4)	0.135 (7)	0.028 (3)	0.014 (4)	0.006 (4)
F1A	0.095 (10)	0.15 (2)	0.23 (3)	0.094 (15)	0.062 (17)	0.12 (2)
F2A	0.069 (11)	0.075 (12)	0.33 (5)	0.041 (9)	−0.09 (2)	−0.032 (18)
F3A	0.137 (12)	0.20 (2)	0.152 (13)	0.107 (15)	0.075 (9)	0.028 (14)
F1B	0.078 (6)	0.094 (10)	0.26 (2)	0.058 (7)	−0.002 (10)	−0.056 (12)
F2B	0.132 (9)	0.257 (19)	0.150 (9)	0.149 (12)	−0.068 (7)	−0.062 (10)
F3B	0.060 (7)	0.130 (11)	0.194 (15)	0.047 (7)	0.047 (8)	0.054 (12)

### Geometric parameters (Å, º)

**Table:**

O4—C13	1.359 (5)	C13—C8	1.405 (5)
O4—C15	1.439 (4)	C10—C9	1.376 (5)
O1—C6	1.299 (4)	C10—C11	1.380 (6)
O1—H1	0.8200	C10—C14	1.478 (7)
N1—C7	1.295 (5)	C11—C12	1.389 (6)
N1—C8	1.426 (5)	C11—H11	0.9300
N2—O3	1.227 (4)	C5—H5	0.9300
N2—O2	1.230 (4)	C9—C8	1.378 (5)
N2—C3	1.443 (5)	C9—H9	0.9300
C7—C1	1.428 (5)	C12—H12	0.9300
C7—H7	0.9300	C15—H15A	0.9600
C1—C2	1.402 (5)	C15—H15B	0.9600
C1—C6	1.428 (5)	C15—H15C	0.9600
C6—C5	1.411 (5)	C14—F1A	1.225 (17)
C4—C5	1.360 (6)	C14—F2A	1.228 (19)
C4—C3	1.397 (5)	C14—F3A	1.481 (17)
C4—H4	0.9300	C14—F1B	1.267 (12)
C2—C3	1.368 (5)	C14—F3B	1.295 (13)
C2—H2	0.9300	C14—F2B	1.379 (12)
C13—C12	1.383 (5)		
			
C13—O4—C15	117.4 (3)	C12—C11—H11	119.5
C6—O1—H1	109.5	C4—C5—C6	121.0 (4)
C7—N1—C8	124.3 (4)	C4—C5—H5	119.5
O3—N2—O2	122.0 (4)	C6—C5—H5	119.5
O3—N2—C3	119.2 (4)	C10—C9—C8	120.7 (4)
O2—N2—C3	118.8 (4)	C10—C9—H9	119.6
N1—C7—C1	121.0 (4)	C8—C9—H9	119.6
N1—C7—H7	119.5	C9—C8—C13	119.5 (4)
C1—C7—H7	119.5	C9—C8—N1	124.6 (4)
C2—C1—C6	119.1 (4)	C13—C8—N1	115.8 (4)
C2—C1—C7	120.0 (4)	C13—C12—C11	119.1 (4)
C6—C1—C7	120.9 (4)	C13—C12—H12	120.4
O1—C6—C5	119.7 (4)	C11—C12—H12	120.4
O1—C6—C1	121.9 (4)	O4—C15—H15A	109.5
C5—C6—C1	118.4 (4)	O4—C15—H15B	109.5
C5—C4—C3	120.3 (4)	H15A—C15—H15B	109.5
C5—C4—H4	119.9	O4—C15—H15C	109.5
C3—C4—H4	119.9	H15A—C15—H15C	109.5
C3—C2—C1	120.5 (4)	H15B—C15—H15C	109.5
C3—C2—H2	119.8	F1A—C14—F2A	111.5 (16)
C1—C2—H2	119.8	F1A—C14—F3A	100.7 (13)
C2—C3—C4	120.7 (4)	F2A—C14—F3A	100.8 (18)
C2—C3—N2	119.8 (4)	F1B—C14—F3B	110.7 (11)
C4—C3—N2	119.5 (4)	F1B—C14—F2B	103.7 (10)
O4—C13—C12	124.8 (4)	F3B—C14—F2B	102.1 (10)
O4—C13—C8	115.2 (4)	F1A—C14—C10	115.9 (11)
C12—C13—C8	120.0 (4)	F2A—C14—C10	117.9 (11)
C9—C10—C11	119.6 (4)	C10—C14—F3A	107.1 (8)
C9—C10—C14	120.3 (5)	F1B—C14—C10	117.5 (8)
C11—C10—C14	120.1 (5)	F3B—C14—C10	111.3 (8)
C10—C11—C12	121.0 (4)	F2B—C14—C10	110.2 (6)
C10—C11—H11	119.5		
			
C8—N1—C7—C1	−177.0 (3)	C14—C10—C9—C8	−178.1 (5)
N1—C7—C1—C2	177.7 (4)	C10—C9—C8—C13	0.6 (6)
N1—C7—C1—C6	−0.3 (6)	C10—C9—C8—N1	177.7 (4)
C2—C1—C6—O1	180.0 (4)	O4—C13—C8—C9	177.8 (4)
C7—C1—C6—O1	−1.9 (6)	C12—C13—C8—C9	−1.0 (6)
C2—C1—C6—C5	0.5 (6)	O4—C13—C8—N1	0.4 (5)
C7—C1—C6—C5	178.5 (4)	C12—C13—C8—N1	−178.4 (3)
C6—C1—C2—C3	0.0 (6)	C7—N1—C8—C9	3.1 (6)
C7—C1—C2—C3	−178.1 (4)	C7—N1—C8—C13	−179.6 (4)
C1—C2—C3—C4	−0.3 (6)	O4—C13—C12—C11	−177.7 (4)
C1—C2—C3—N2	179.8 (4)	C8—C13—C12—C11	0.9 (6)
C5—C4—C3—C2	0.2 (6)	C10—C11—C12—C13	−0.4 (7)
C5—C4—C3—N2	−179.9 (4)	C9—C10—C14—F1A	136 (2)
O3—N2—C3—C2	−0.7 (6)	C11—C10—C14—F1A	−42 (2)
O2—N2—C3—C2	−177.9 (4)	C9—C10—C14—F2A	0 (3)
O3—N2—C3—C4	179.4 (4)	C11—C10—C14—F2A	−178 (3)
O2—N2—C3—C4	2.2 (6)	C9—C10—C14—F1B	−179.0 (14)
C15—O4—C13—C12	0.5 (6)	C11—C10—C14—F1B	2.9 (16)
C15—O4—C13—C8	−178.3 (4)	C9—C10—C14—F3B	−49.9 (13)
C9—C10—C11—C12	−0.1 (7)	C11—C10—C14—F3B	132.0 (12)
C14—C10—C11—C12	178.0 (5)	C9—C10—C14—F2B	62.5 (13)
C3—C4—C5—C6	0.3 (6)	C11—C10—C14—F2B	−115.5 (12)
O1—C6—C5—C4	179.8 (4)	C9—C10—C14—F3A	−112.9 (16)
C1—C6—C5—C4	−0.6 (6)	C11—C10—C14—F3A	69.1 (16)
C11—C10—C9—C8	0.0 (7)		

### Hydrogen-bond geometry (Å, º)

**Table:**

*D*—H···*A*	*D*—H	H···*A*	*D*···*A*	*D*—H···*A*
O1—H1···N1	0.82	1.84	2.571 (4)	148
O1—H1···O4	0.82	2.76	3.468 (4)	146
C7—H7···O2^i^	0.93	2.55	3.476 (7)	176
C9—H9···O2^i^	0.93	2.46	3.378 (6)	169

Symmetry code: (i) −*y*+4/3, *x*−*y*−1/3, *z*−1/3.

## References

[bb1] Faridbod, F., Ganjali, M. R., Dinarvand, R., Norouzi, P. & Riahi, S. (2008). *Sensors*, **8**, 1645–1703. 10.3390/s8031645 PMC3663017 27879786

[bb2] Farrugia, L. J. (2012). *J. Appl. Cryst.* **45**, 849–854.

[bb3] Hadjoudis, E., Vittorakis, M. & Moustakali-Mavridis, I. (1987). *Tetrahedron*, **43**, 1345–1360.

[bb4] Karadayı, N., Albayrak, Ç., Odabaşoğlu, M. & Büyükgüngör, O. (2006). *Acta Cryst.* E**62**, o1699–o1701.

[bb5] Karadayı, N., Gözüyeşil, S., Güzel, B., Kazak, C. & Büyükgüngör, O. (2003). *Acta Cryst.* E**59**, o851–o853.

[bb6] Karadayı, N., Köysal, Y., Şahin, S., Coşkun, E. & Büyükgüngör, O. (2013). *Acta Cryst.* E**69**, o889. 10.1107/S1600536813012518 PMC3685046 23795065

[bb7] Raja, K. K., Bilal, I. M., Thambidurai, S., Rajagopal, G. & SubbiahPandi, A. (2008). *Acta Cryst.* E**64**, o2265. 10.1107/S1600536808035071 PMC2959941 21581246

[bb8] Santos, M. L. P., Bagatin, I. A., Pereira, E. M. & Ferreira, A. M. D. C. (2001). *J. Chem. Soc. Dalton Trans.* pp. 838–844.

[bb9] Sheldrick, G. M. (2008). *Acta Cryst.* A**64**, 112–122. 10.1107/S0108767307043930 18156677

[bb10] Stoe & Cie (2002). *X-AREA* and *X-RED32*. Stoe & Cie, Darmstadt, Germany.

[bb11] Tarafder, M. T. H., Chew, K., Crouse, K. A., Ali, A. M., Yamin, B. M. & Fun, H.-K. (2002). *Polyhedron*, **21**, 2683–2690.

