# Supplementary figures and images for: Crystal structure of 2-{[2-meth­oxy-5-(tri­fluoro­meth­yl)phen­yl]iminomethyl}-4-nitro­phenol
Source: Acta Crystallogr E Crystallogr Commun. 2015 Jun 13;71(Pt 7):o466–7. doi: 10.1107/S2056989015010129 (PMC4518952; doi:10.1107/S2056989015010129)

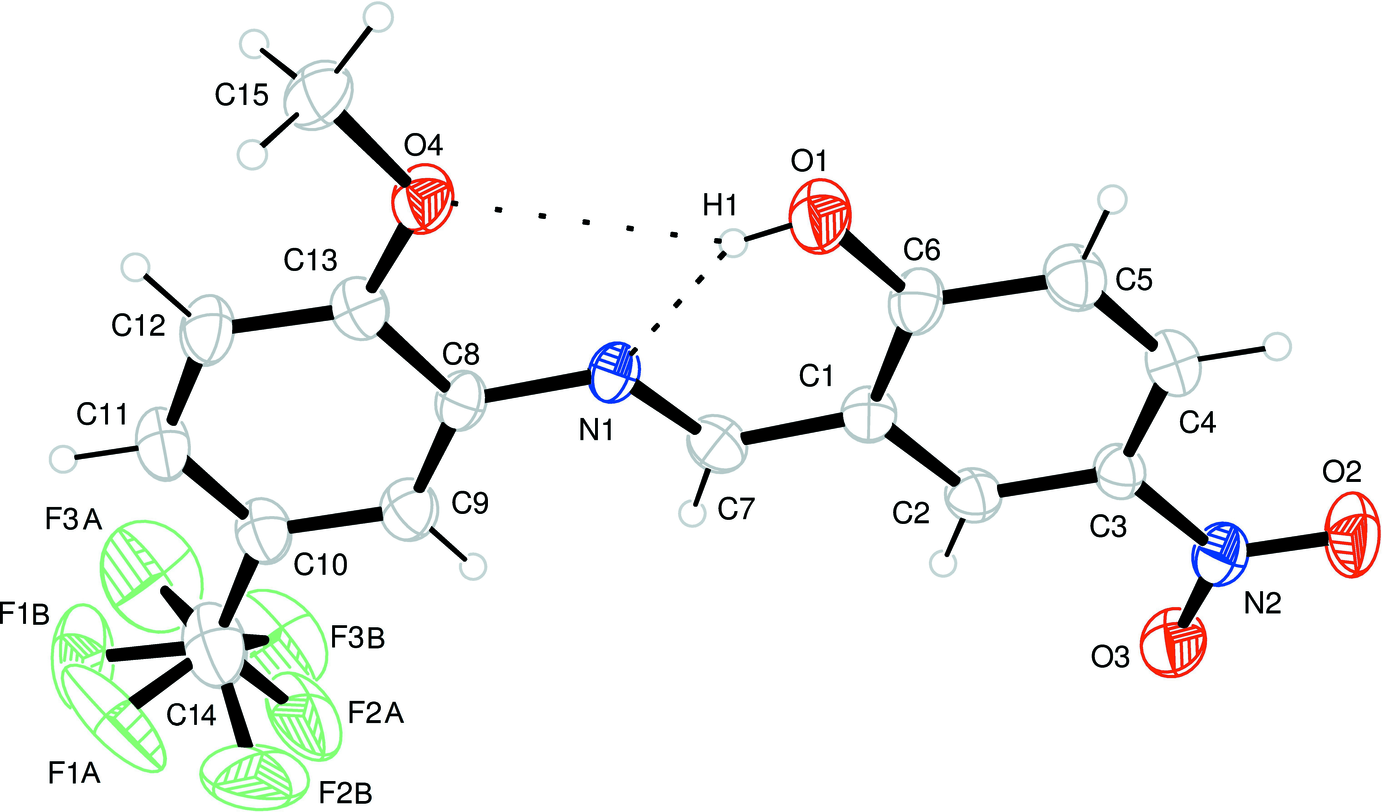

Supplement: Supplementary file 4 [file e-71-0o466-fig1.tif]
